# Gut and intestinal biometrics of the giant trevally, *Caranx ignobilis,* fed an experimental diet with difference sources of activated charcoal

**DOI:** 10.12688/f1000research.23788.2

**Published:** 2020-10-13

**Authors:** Firdus Firdus, Samadi Samadi, Abdullah A. Muhammadar, Muhammad A. Sarong, Zainal A. Muchlisin, Widya Sari, Agung S. Batubara

**Affiliations:** 1 Departement of Biology, Faculty of Mathematics and Natural Science, Universitas Syiah Kuala, Banda Aceh, Aceh, 23111, Indonesia; 2 Graduate School of Mathematics and Applied Science, Universitas Syiah Kuala, Banda Aceh, Aceh, 23111, Indonesia; 3 Animal Husbandry Department, The Faculty of Agriculture, Syiah Kuala University, Banda Aceh, Aceh, 23111, Indonesia; 4 Departement of Aquaculture, Faculty of Marine and Fishery, Universitas Syiah Kuala, Banda Aceh, Aceh, 23111, Indonesia; 5 Departement of Biology Education, Faculty of Teacher Training and Education, Universitas Syiah Kuala, Banda Aceh, Aceh, 23111, Indonesia

**Keywords:** Foveola gastrica, villous intestine, coconut shell, mangrove wood, rice husk, and kernel palm shell

## Abstract

**Background** **:** The giant trevally, *Caranx ignobilis*, is a commercially important marine fish in Indonesia. This species was initially cultured in Aceh Province. Previous reports showed that charcoal has a positive effect on survival and feed utilization of the giant trevally. However, the effects of adding charcoal to the diet on gut and intestine biometrics has, to our knowledge, never been described.

**Methods** **:** Four activated charcoal sources were tested in this study using a completely randomized experimental design; coconut shell charcoal, mangrove wood charcoal, rice husk charcoal, and kernel palm shell charcoal. All treatments were performed with four replications. Juvenile giant trevally (average body weight, 16.52 ± 3.12 g; and average total length, 10.26 ± 0.64 cm) were stocked into the experimental tank at a density of 15 fish per tank. The fish were fed an experimental diet twice daily at 7 AM and 5 PM *ad satiation* for 42 days.

**Results** **:** Analysis of variance showed that adding charcoal to the diet had significant effects on the length and width of the foveola gastrica and villous intestine (P < 0.05). The greatest length and width of the foveola gastrica was recorded in fish fed an experimental diet of rice husk charcoal with average values of 311.811 ± 9.869 µm and 241.786 ± 10.394 µm, respectively. The greatest length of intestinal villous was found in fish fed the mangrove wood charcoal diet, with a value of 135.012 ± 5.147 µm, but this length was not significantly different to that in fish fed rice charcoal and kernel palm shell charcoal. However, the greatest width of intestinal villous was recorded in fish fed the control diet (without charcoal; P < 0.05).

**Conclusion:** The optimal sizes of the foveola gastrica and villous intestine were found in fish fed an experimental diet with rice husk charcoal.

## Introduction

Trevally fish are a commercially important group of marine fish in the family Carangidae. A total of 146 species of trevally have been recorded worldwide ^1^. These fish are distributed in tropical, subtropical, and temperate waters ^2– 7^. In Indonesia, trevally fish are found in the Aceh waters ^8, 9^, East Borneo ^10^, Papua and Wester Nusa Tenggara ^11, 12^, and Java ^13^.

Giant trevally, *Caranx ignobilis,* is among the most popular trevally fish in Indonesia. The population of this species has declined over the years due to overfishing ^7, 14– 16^. Culture of this fish has been initiated in Aceh Province, Indonesia. However, farmers are faced with a feeding obstacle. Giant trevally in culture systems are currently fed waste fish and a commercial diet (Hi-Pro-Vite, Central Proteina Prima Company). The commercial diet is costly and difficult to obtain in remote areas, and the waste fish supply is very seasonal. Trash fish are limited in nutrients, particularly the essential amino acid composition ^17^. Therefore, it is crucial to formulate a diet for giant trevally using local raw materials with higher protein, that is inexpensive, easy to find, and digestible.

Activated charcoal is commonly added to the diet to increase digestibility and trigger growth in fish. For example, Jahan *et al*. ^18^ successfully used activated charcoal to increase the digestibility and growth performance of river catfish, *Pangasiaodon* sp. Other researchers have used charcoal in the diets of fish species, such as Nile tilapia, *Oreochromis niloticus* ^19– 21^, tiger pufferfish, *Takifugu rubripes* ^22^, Japanese flounder, *Paralichthys olivaceus* ^23^, African catfish, *Clarias gariepinus* ^24, 25^, gilthead seabream, *Sparus aurata* ^26^, and sturgeon, *Huso huso* ^27^. Firdus *et al*. ^28^ added rice husk charcoal to the diet of giant trevally. However, the effect of charcoal on the morphology of the gut and intestine has not been reported.

Organogenesis of the digestive system occurs as fish age, and this process is strongly dependent on the quantity and quality of food ^29– 32^, which is related to the development of mucosal cells, amplification of apical plasma membranes, and formation of the foveola gastrica and intestinal villi ^33, 34^. It has been hypothesized that adding activated charcoal to the diet triggers the digestive organogenesis system process ^35, 36^. In this study, we tested four charcoal sources in the diet to evaluate the morphology of the gut and intestine of giant trevally. Information on the gut and intestinal morphology is important to understand the absorption mechanism of nutrients from the diet.

## Methods

### Time and site

The study was conducted at the Center for Brackish Water Aquaculture, Ujung Batee, Aceh, Indonesia from February to July 2018. The activated charcoal was characterized at the Integrated Laboratory of Calibration, Universitas Gajah Mada, Yogyakarta, Indonesia. Histological samples were prepared at the Laboratory of Histology, Faculty of Mathematics and Natural Sciences, Universitas Syiah Kuala, Banda Aceh, Indonesia.

### Experimental design

A completely randomized experimental design with five treatments consisting of control and four different charcoal sources was used in this study. The experimental groups were: (A) the experimental diet without charcoal, (B) the experimental diet with 2% charcoal from coconut shell, (C) the experimental diet with 2% charcoal from mangrove wood, (D) the experimental diet with 2% charcoal from rice husk, and (E) the experimental diet with 2% charcoal from kernel palm shell. All treatments were performed with four replications.

### Experimental fish

A total of 300 giant trevally juveniles of mixed sex (average body weight, 16.52 ± 3.12 g; total length, 10.28 ± 0.64 cm) were purchased from a local farmer in Lancang Barat Village, Aceh Utara District, Aceh, Indonesia. The fish were acclimatized in ponds (ponds size 2 m x 1.8 m and temperature of around 29°C) at the Center for Brackish Water Aquaculture, Ujung Batee for 2 weeks. The fish were fed an experimental diet containing 50% crude protein twice daily at 7 AM and 5 PM at 3% of body weight per day ( Table 1).

**Table 1.** The composition of raw materials in the experimental diet (g kg ^−1^) with 50% crude protein.

Raw materials	Crude protein (%)	Composition (g kg ^−1^)	
		Diet without charcoal (Diet A, Control)	Diet with charcoal (Diet B, C, D, E)
Ebi-shrimp meal	58.80	50	50
Fish meal	59.00	660	660
Rice flour	7.26	180	160
Soybean meal	45.06	20	20
Bloodmeal	71.00	20	20
Corn flour	6.48	10	10
Coconut oil	0	5	5
CaCO3	0	5	5
Isoleucine	100	10	10
L-Tryptophan	100	17.5	17.5
DL-Methionine	100	17.5	17.5
Mineral mix	0	5	5
Active charcoal	0	-	20
Total material		1000	1000
Total crude protein		50%	50%

Note: (A) diet without charcoal, (B) diet with charcoal from coconut shells, (C) diet with charcoal from mangrove wood, (D) diet with charcoal from rice husks, (E) diet with charcoal from kernel palm shells.

### Charcoal preparation and activation

The raw coconut shells, mangrove wood, rice husks, and kernel palm shells were chopped and ground. Approximately 500 g of the ground materials were placed on aluminum foil and heated in a furnace at 400°C for 1 hour. Nitrogen gas was flowed into the furnace to remove the oxygen. Then, the temperature was decreased to 30°C gradually and held for 1 hour. After 1 hour, the charcoal was removed from the furnace, sieved through a No. 40 mesh, and held in a jar before activating. A total of 100 g of sieved charcoal was taken and mixed with 400 ml of 0.2 M citric acid. The solution was stirred for 24 hours. After 24 hours, the solution was filtered through filter paper. The filtered charcoal was washed with distilled water and dried in an oven at 110°C for 24 hours.

### Diet preparation

The experimental diet was formulated from both plant and animal-based protein sources, such as Ebi-shrimp meal, fish meal, blood meal, soybean meal, rice flour, and corn flour. All raw materials were subjected to a proximate analysis before use in the formulation. Three types of amino acids i.e. isoleucine, L-tryptophan, and DL-methionine were also added ( Table 1). A total of 2% of the tested charcoal sources was added to the formulation ( Table 1). The formulated diets were subjected to a proximate analysis before use in the experiment.

### Stocking and feeding

The fish was captured randomly, measured for body weight and total length, and then distributed into 20 plastic containers (48 × 43 × 70 cm) at a stocking density of 15 fish per container. The water volume in the container was 75 L. The fish were fed an experimental diet twice daily at 7 AM and 5 PM to satiation for 42 days.

### Histological sample preparation

Gastric and intestinal samples were collected at the end of the study. Three fish from each treatment were taken randomly from the experimental tanks. The fish were anesthetized with 30 mg L ^−1^ clove oil ^37^, and the abdomen of the fish was gently dissected following the procedure of Purushothaman *et al*. ^38^. The stomach and intestines were removed with scalpel scissors and preserved in 4% formalin for 1 week. Histological sampling was carried using the paraffin method based on Osman and Caceci ^39^. The samples were dehydrated through an alcohol series and cleared in xylol. Subsequently, the gut and intestine samples were embedded in paraffin. The paraffin block was sectioned to 6 µm, and the sections were stained with hematoxylin and eosin. The size (height and width) of villi was determined using a binocular microscope (Zeiss Primo Star, Carl Zeiss Suzhou Co., Ltd., Suzhou, China) which was connected to a CCD camera and computer monitor ^19^. All efforts were made to lessen harm to the animals by complying to the guidelines of ethics animal use in research of Syiah Kuala University.

### Data analysis

The qualitative gut and intestinal morphology data were subjected to one-way analysis of variance followed by Duncan’s multiple range test. The analysis was performed using SPSS ver. 18.0 software. The qualitative (histological) gut and intestinal data were analyzed descriptively. A *P*-value < 0.05 was considered significant.

## Results

**Figure 1.**
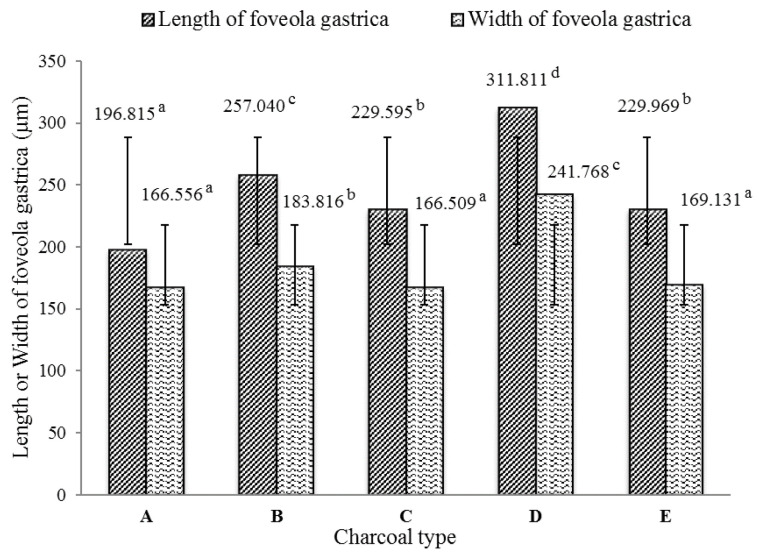
The average of length and width of the *foveola gastrica*. ( **A**) Diet without charcoal, ( **B**) diet with coconut shell charcoal, ( **C**) diet with mangrove wood charcoal, ( **D**) diet with rice husk charcoal, ( **E**) diet with kernel palm shell charcoal.

**Figure 2.**
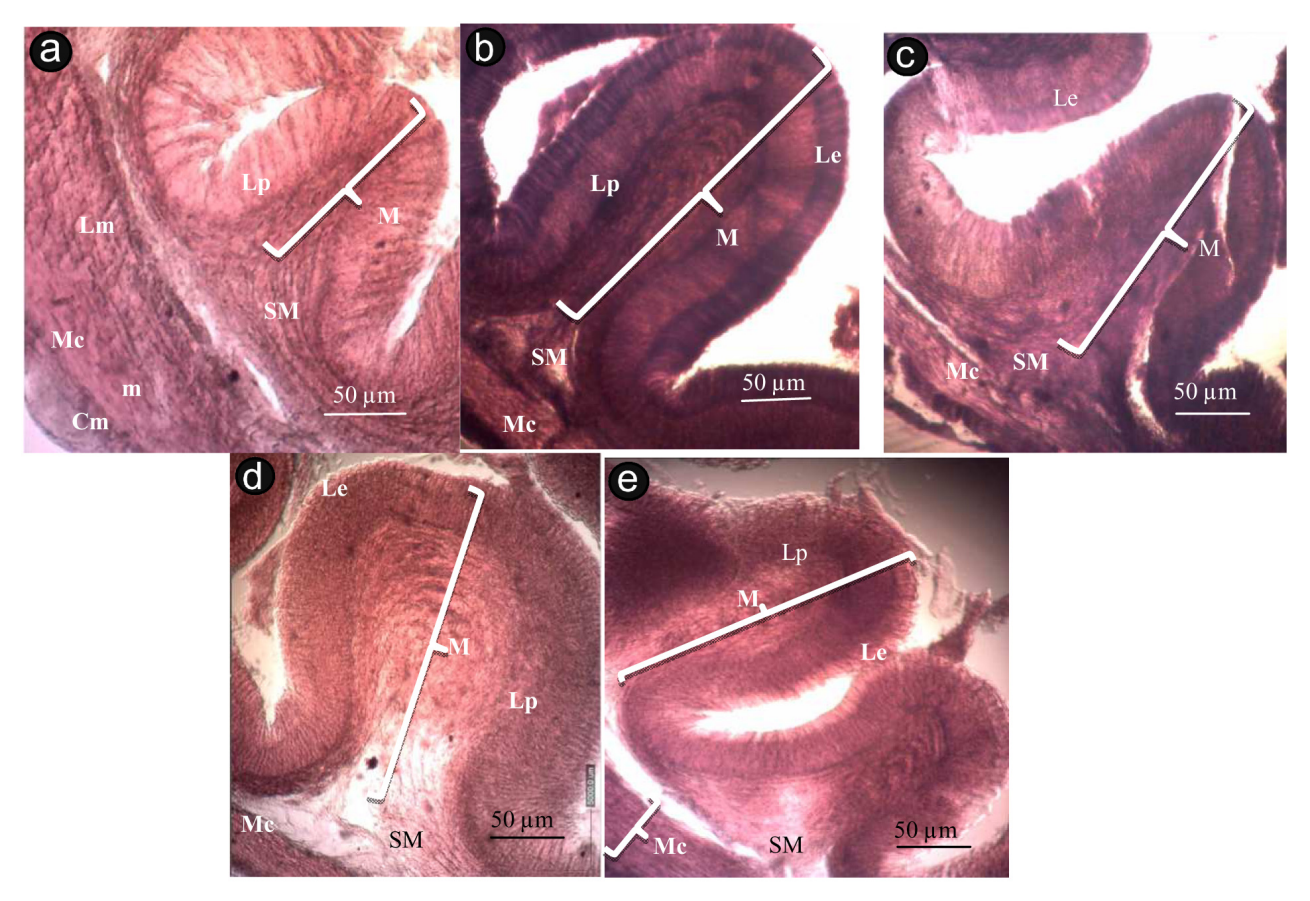
Histology of *foveola gastrica* from a juvenile giant trevally. ( **A**) Diet without charcoal, ( **B**) diet with coconut shell charcoal, ( **C**) diet with mangrove wood charcoal, ( **D**) diet with rice husk charcoal, ( **E**) diet with kernel palm shell charcoal. M, tunica mucosa; SM, tunica submucosa; Mc, tunica muscularis; Le, lamina epithelialis; Lp, lamina propria; m, muscle; Lm, longitudinal muscle fibers; Cm, circular muscle fibers (Cm).

Adding activated charcoal to the diet significantly affected the length and width of the foveola gastrica and intestinal villi ( *P* < 0.05). In general, fish fed the activated charcoal diets produced better results than those not fed the charcoal ( Figure 1 and Figure 2). The best foveola gastrica morphology was obtained with the rice husk charcoal and the mean length and width of the foveola gastrica were 311.811 µm and 241.786 µm, respectively; followed by coconut shell charcoal (257.040 µm and 183.816 µm), kernel palm charcoal (229.969 µm and 169.131 µm µm), and mangrove wood charcoal (229.595 µm and 166.509 µm).

**Figure 3.**
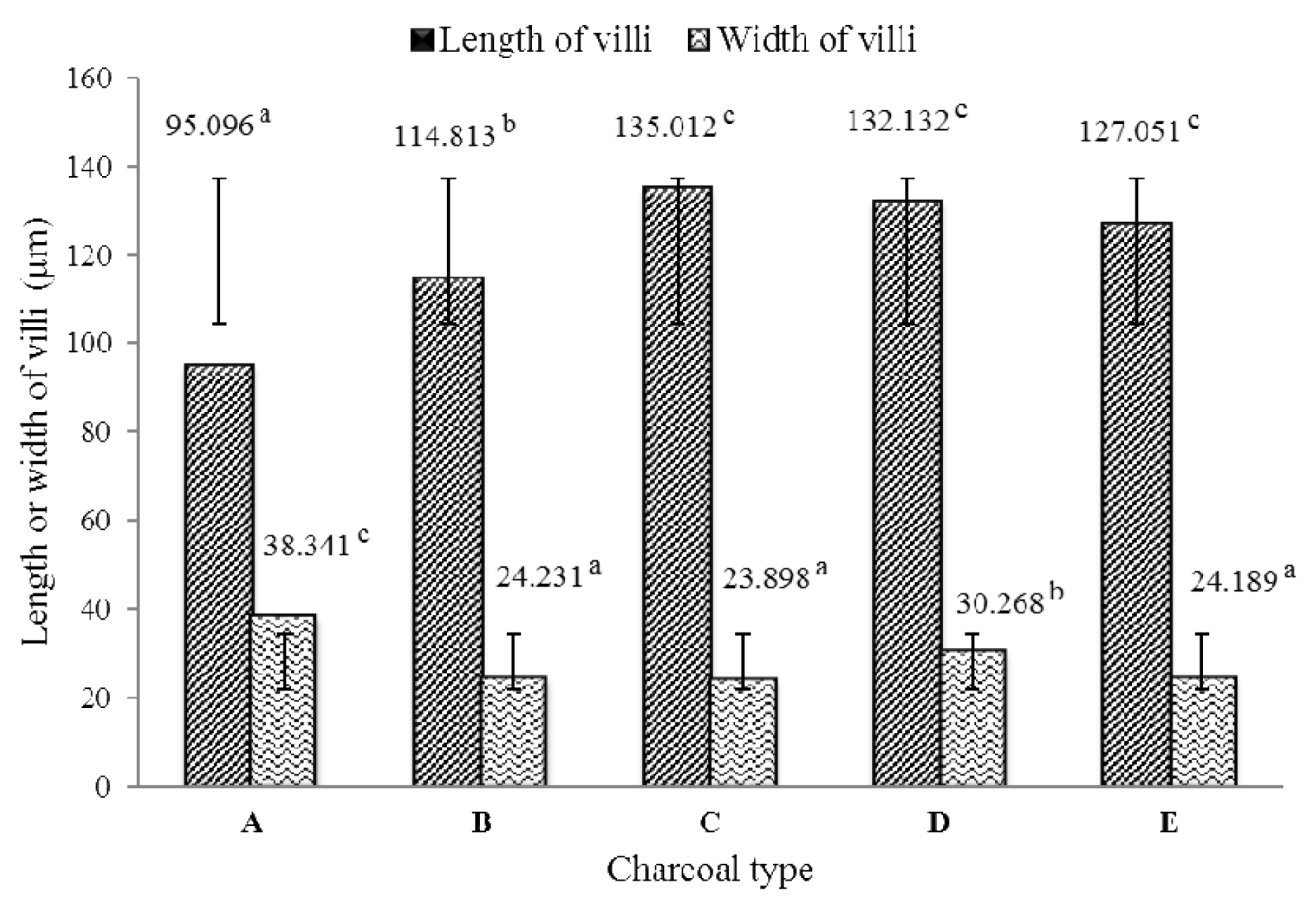
The average length and width of intestine villi from juvenile giant trevally. ( **A**) Diet without charcoal, ( **B**) diet with coconut shell charcoal, ( **C**) diet with mangrove wood charcoal, ( **D**) diet with rice husk charcoal, ( **E**) diet with kernel palm shell charcoal.

**Figure 4.**
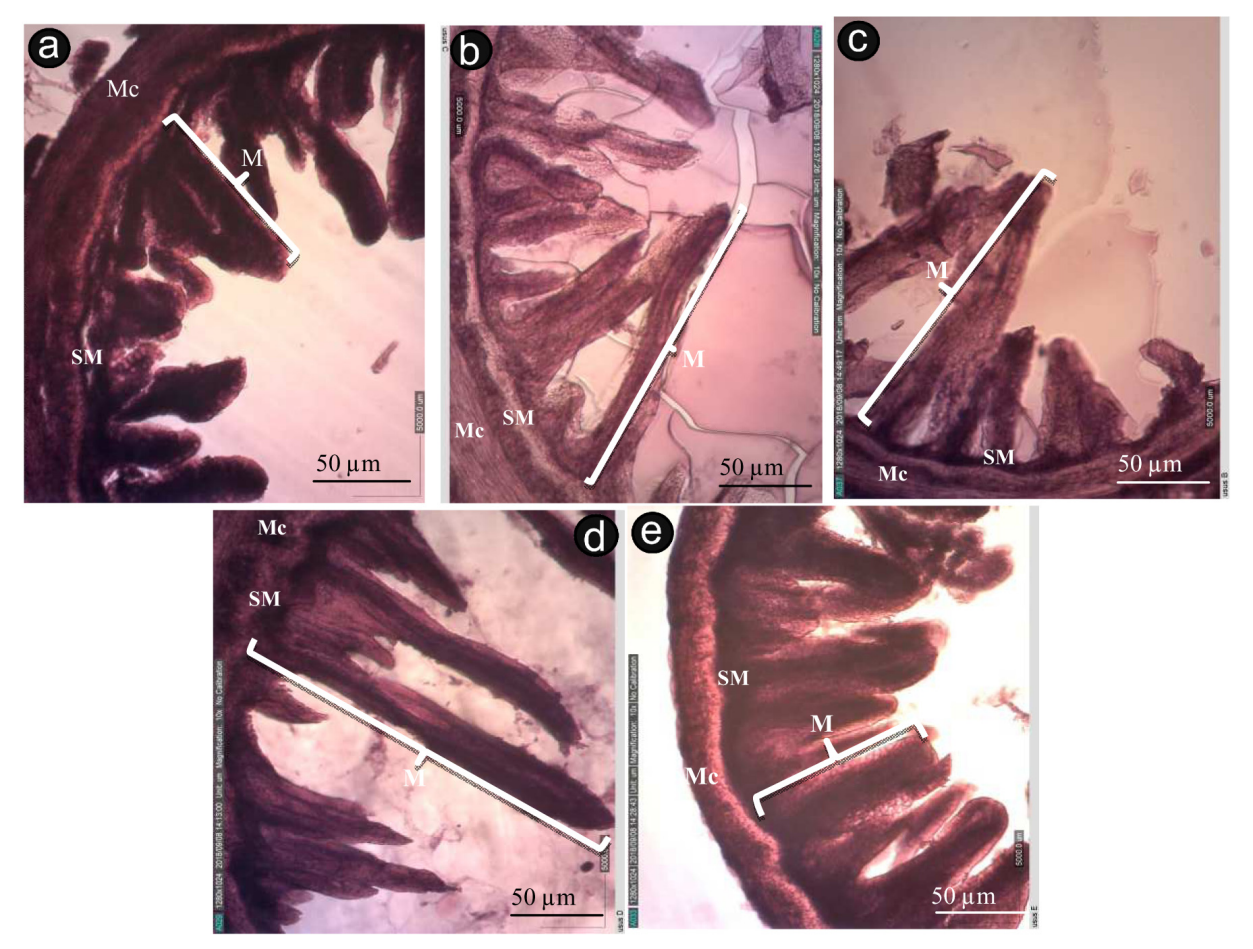
Histology of intestinal villi from a giant trevally juvenile. ( **A**) Diet without charcoal, ( **B**) diet with coconut shell charcoal, ( **C**) diet with mangrove wood charcoal, ( **D**) diet with rice husk charcoal, ( **E**) diet with kernel palm shell charcoal. M, tunica mucosa; SM, tunica submucosa; Mc, tunica muscularis.

The greatest length of the villous intestine was recorded in fish fed a diet with activated charcoal than those not fed the activated charcoal ( Figure 3). The greatest growth of intestinal villi was determined in the mangrove active charcoal (mean, 135.012 µm) group, but this value was not significantly different from the rice husk or kernel palm shell charcoals ( Figure 4). However, the greatest intestinal villi width was obtained in the treatment without activated charcoal (38.341 µm), and this value was significantly different from the other treatments.

Raw biometic data, in addition to unprocessed imaged, are available as *Underlying data* ^40– 42^.

## Discussion

The results show that adding activated charcoal to the diet of *C. ignobilis* significantly affected favoela gastrica and intestinal villi biometrics. According to Pirarat *et al*. ^19^, activated charcoal plays a significant role stimulating the development of epithelial cells of the digestive organs. Activated charcoal in the diet functions as a decontaminating agent to eliminate pathogenic organisms and toxic compounds, such as mycotoxins ^20^. Hence, a longer foveola gastrica and larger intestinal villi were able to provide more nutrients to be absorbed due to a larger surface area of digestive organs ^43^. Optimal development of the alimentary tract was recorded in giant trevally juveniles fed the experimental diet containing rice husk charcoal. This was presumably due to the high hemicellulose, cellulose, and lignin contents in the rice husk charcoal. A previous report indicated that rice husk charcoal contains 29.3% hemicellulose, 34.4% cellulose, and 19.2% lignin ^44^, while mangrove wood charcoal has 30% hemicellulose, 36% cellulose, and 28% lignin ^45^, coconut shell charcoal has 19.27% hemicellulose, 33.61% cellulose, and 36.51% lignin ^46^, and kernel palm shell charcoal has 26.27% cellulose, 12.61% hemicellulose, and 42.96% lignin ^47^. Maria and Banu ^48^ and Jamilatun *et al*. ^49^ reported that the concentration and quality of charcoal depend on the composition of hemicellulose, cellulose, and lignin. The quality of the activated charcoal is higher when these three components increase. According to Jasman ^50^, rice husk contains 85–95% activated charcoal, while mangrove wood has 76% activated charcoal ^51^, kernel palm shell 65% activated charcoal ^47^, and coconut shell has 60% activated charcoal ^46^.

The microscopic observations showed that the intestinal villi of the fish fed the diet with activated rice husk charcoal had a more pointed shape compared to other treatments, in which the villi tended to be round and blunt. According to Guo *et al*. ^52^, blunt or rounded villi probably occur due to inflammation in the intestinal mucosa, which is characterized by infiltration of neutrophils into the lamina propria. An increase of intestinal villus size is related to nutrient absorption capacity. According to Nafis *et al*. ^53^, long mucosal folds increase nutrient absorption and reduce food flow movement due to reduced peristaltic contractions, which provides sufficient time to optimally absorb nutrients. The increase in intestinal villi size is strongly related to the activities of digestive enzymes, such as lactase, sucrase, alkaline phosphatase, and disaccharidase ^54– 57^.

The morphology of the intestinal villi of fish fed a diet without activated charcoal was wider and shorter than that of fish fed the diets with activated charcoal. This was probably due to impaired intestinal mucosal integrity, causing interference in nutrient absorption. According to Choct ^58^, shortening of the intestinal villi is related to the accumulation of intestinal pathogenic bacteria, resulting in increased susceptibility to infection in the intestinal mucosal layer. This causes the digestive organs to form more secretory cells than absorbent cells, which reduces nutrient uptake ^59, 60^. The active charcoal likely acts as an adsorbent of metabolic pathogens in the intestine in the form of endotoxins and ammonia, therefore, it was able to improve intestinal function ^61^.

## Conclusions

The application of activated charcoal in the diet significantly affected the length and width of the foveola gastrica and intestinal villi of giant trevally, *C. ignobilis*. The optimal biometrics of the foveola gastrica and intestinal villi were observed in fish fed the experimental diet with activated rice husk charcoal.

## Data availability

Figshare: Gut and intestinal biometrics of the giant trevally, Caranx ignobilis, fed an experimental diet with difference sources of activated charcoal. https://doi.org/10.6084/m9.figshare.12203525.v2 ^40^.

This project contains the following underlying data:

- DATA BIOMETRIC GUT OF GIANT TREVALLY Caranx ignobilis_Edited (XLSX). (Raw biometric data for the foveola gastrica of all fish examined in this study.)
- DATA BIOMETRIC OF INTESTINE OF GIANT TREVALLY Caranx ignobilis_edited (XLSX). (Raw biometric data for the intestinal villi of all fish examined in this study.)

Figshare: Gut and intestinal biometrics of the giant trevally, Caranx ignobilis, fed an experimental diet with difference sources of activated charcoal. https://doi.org/10.6084/m9.figshare.12301124.v2 ^41^.

This project contains uncropped, unprocessed images of the intestinal villi of giant trevally.

Figshare: Gut and intestinal biometrics of the giant trevally, Caranx ignobilis, fed an experimental diet with difference sources of activated charcoaltem. https://doi.org/10.6084/m9.figshare.12269606.v2 ^42^.

This project contains uncropped, unprocessed images of the foveola gastrica of the giant trevally.

Data are available under the terms of the Creative Commons Attribution 4.0 International license (CC-BY 4.0).
