# Supplementary material for: Gut and intestinal biometrics of the giant trevally, Caranx ignobilis, fed an experimental diet with difference sources of activated charcoal
Source: F1000Res. 2020 Oct 13;9:444. Originally published 2020 May 26. [Version 2] doi: 10.12688/f1000research.23788.2 (PMC7355220; doi:10.12688/f1000research.23788.2)
Supplement: Peer review history

# Peer review: Reviewer response for version 2

**DOI:** 10.5256/f1000research.28490.r72869

**Published:** 2020-10-13

**Authors:** Abdus Salam

**Affiliations:** 1 Department of Aquaculture, Faculty of Fisheries, Bangladesh Agricultural University, Mymensingh, Bangladesh

Gut and intestinal biometrics of the giant trevally, *Caranx ignobilis,* fed an experimental diet with different sources of activated charcoal.

 The title should be different not difference. Others are fine.

Is the work clearly and accurately presented and does it cite the current literature?

Yes

If applicable, is the statistical analysis and its interpretation appropriate?

Yes

Are all the source data underlying the results available to ensure full reproducibility?

Yes

Is the study design appropriate and is the work technically sound?

Yes

Are the conclusions drawn adequately supported by the results?

Yes

Are sufficient details of methods and analysis provided to allow replication by others?

Yes

Reviewer Expertise:

Aquaculture, Aquaponics, fish nutrition

I confirm that I have read this submission and believe that I have an appropriate level of expertise to confirm that it is of an acceptable scientific standard.

# Peer review: Reviewer response for version 1

**DOI:** 10.5256/f1000research.26249.r65152

**Published:** 2020-07-10

**Authors:** Abdus Salam

**Affiliations:** 1 Department of Aquaculture, Faculty of Fisheries, Bangladesh Agricultural University, Mymensingh, Bangladesh

- It seems the manuscript has potentials in the scientific world, and work was done and formatted with care. The abstract is concise but well written. 
- The introduction has supported with up to date literature and aim and objectives are well described. 
- Results supported with details and tables and figures were provided. 
- The discussion was also well described and literature supported.

Is the work clearly and accurately presented and does it cite the current literature?

Yes

If applicable, is the statistical analysis and its interpretation appropriate?

Yes

Are all the source data underlying the results available to ensure full reproducibility?

Yes

Is the study design appropriate and is the work technically sound?

Yes

Are the conclusions drawn adequately supported by the results?

Yes

Are sufficient details of methods and analysis provided to allow replication by others?

Yes

Reviewer Expertise:

Aquaculture

I confirm that I have read this submission and believe that I have an appropriate level of expertise to confirm that it is of an acceptable scientific standard.

# Peer review: Reviewer response for version 1

**DOI:** 10.5256/f1000research.26249.r63939

**Published:** 2020-06-15

**Authors:** Srikanta Samanta

**Affiliations:** 1 Principal Scientist & Head (Acting) Riverine Ecology and Fisheries Division, ICAR-Central Inland Fisheries Research Institute, Barrackpore, India

The paper has communicated a short but interesting study on the beneficial effects of adding different types of charcoal as a dietary component of giant trevally. The addition of charcoal has significantly improved the gastro-intestinal microstructures including foveola gastrica & villous intestine and thereby, has the potentiality to improve the fish nutrition and health. The rice husk was recorded as the best source of charcoal with respect to 3 other sources including coconut shell, mangrove wood, and kernel palm shell. However, some deficiencies have been noticed which needs rectification.

1. In Abstract : This species was---: Better to use The species was---
2. Four activated charcoal sources were tested in this study -----but in Experimental Design mentioned ----A completely randomized experimental design with five different charcoal sources----
3. In Abstract : average total length, 10.26 ± 0.64 cm, In Experimental fish : total length, 10.28 ± 0.64 cm
4. ponds size 2 x 1.8 m. No unit given after 2. It may be 2 m X 1.8 m
5. (see Table 1) : see may be deleted
6. Table 1 : CaCo3 : CaCO3
7. In Abstract :  and 241.786 μm --- In Figure 1 241.768
8. Figure 2 and 4 : Histological sample ---- may be replaced by ---- Histology
9. Sentence to be modified : Hence, a longer foveola gastrica and larger intestinal villi provide a larger surface area to absorb nutrients43.
10. Sentence to be modified : A previous report indicated that rice husk charcoal contains 39% hemicellulose, 44% cellulose, and 30% lignin44 : Since the addition of 3 parameters are giving values >100%, you may mention "up to" otherwise people will not understand.
11. Sentence to be modified/changed : The active charcoal likely acts as an adsorbent of metabolic pathogens in the intestine in the form of endotoxins and ammonia to improve intestinal function61. The sentence to be rewritten to increase clarity.
12. It has been noticed that the SE values have gone out of the bar area. Please check about it and reason behind. For the paper, the fish growth parameters may be given as supplements for better clarity.

Is the work clearly and accurately presented and does it cite the current literature?

Yes

If applicable, is the statistical analysis and its interpretation appropriate?

Yes

Are all the source data underlying the results available to ensure full reproducibility?

Yes

Is the study design appropriate and is the work technically sound?

Yes

Are the conclusions drawn adequately supported by the results?

Yes

Are sufficient details of methods and analysis provided to allow replication by others?

Yes

Reviewer Expertise:

Aquatic Chemistry

I confirm that I have read this submission and believe that I have an appropriate level of expertise to confirm that it is of an acceptable scientific standard.

# Response

**Published:** 2020-07-22

**Authors:** Samadi Samadi

**Affiliations:** Universitas Syiah Kuala, Indonesia

Dear Prof. Srikanta Samanta,

 Thank you very much for your comment and we will revise the article based on your suggestions.

 Best regards,

 Samadi
